# Direct Hot Solid–Liquid Extraction (DH-SLE): A High-Yield Greener Technique for Lipid Recovery from Coffee Beans

**DOI:** 10.3390/plants14020185

**Published:** 2025-01-11

**Authors:** Daliane Cláudia de Faria, Maria Eliana Lopes Ribeiro de Queiroz, Fábio Junior Moreira Novaes

**Affiliations:** Departamento de Química, Universidade Federal de Viçosa, Campus Universitário, Avenida Peter Henry Rolfs, s/n, Viçosa 36570-900, MG, Brazil; daliane.faria@ufv.br (D.C.d.F.); meliana@ufv.br (M.E.L.R.d.Q.)

**Keywords:** green chemistry, *Coffea arabica*, lipids, gas chromatography, mass spectrometry, 2030 Agenda

## Abstract

Soxhlet extraction is a method recommended by the Association of Official Analytical Chemists (AOAC) to determine the lipid content in plant samples. Generally, n-hexane (toxicity grade 5) is used as the solvent (≈300 mL; ≈30 g sample) at boiling temperatures (69 °C) for long times (≤16 h) under a chilled water reflux (≈90 L/h), proportionally aggravated by the number of repetitions and samples determined. In this sense, the technique is neither safe nor sustainable for the analyst or the environment. This article presents the development of an alternative and more sustainable procedure for determining the lipid content in raw Arabica coffee beans. A 3^3^ full factorial design was used to perform direct hot solid–liquid extractions in 4 mL vials, varying the ground grains and solvent ratios, temperatures, and times. An optimal condition resulted in an extractive yield statistically equivalent to Soxhlet, without variation in the composition of the oil fatty acids determined by GC-MS after hole oil transesterification. This procedure was presented as a sustainable alternative to Soxhlet extraction because it does not require water for cooling and needs a smaller volume of solvent (2 mL) and sample mass (0.2 g); it also has a smaller generated residue, as well as requiring a shorter time (1.5 h) and less energy expenditure for extraction.

## 1. Introduction

Coffee is one of the most important products in the global food chain, generating around USD 200 billion annually [1]. The beverage is enjoyed by 80% of the world’s adult population, with the Arabica species (*Coffea arabica* L., Rubiaceae) being the most cultivated (75.4% of the total) and of greatest commercial value for producing a beverage rich in aromas and flavors [2,3,4].

Raw coffee beans contain about 800 non-volatile substances, such as polysaccharides, lipids (10–17%) [5,6,7], caffeine (0.2–0.4%) [7,8,9], chlorogenic acids (3.18–7.9%) [7,10,11,12], trigonelline (0.98–2.0) [7], sucrose (8.34%) [4,13], caffeic acid, nicotinic acid [14], proteins (11–15%) [7], and amino acids [15], among others. This complex chemical composition of coffee is fundamental to its flavor, aroma, and overall quality after the roasting process, where around 1000 volatile organic compounds are formed and released [16,17]. Some of these compounds are perceptible to coffee roast masters and certified panelists (Q-graders) who ensure the sensory evaluation and price of the sample [4,18].

Lipids are a significant class in raw coffee beans (7–17% *w*/*w*), composed mainly of triacylglycerols (75–96%), esterified (2.7–18.5%) and free (0.2–0.6%) cafestol and kahweol diterpenes, di- and monoacylglycerols (1.1–3.3%), free fatty acids (1.0–11%), sterols (0.5–2.2%), hydrocarbons (0.7–2.2%), caffeine (0.2–0.4%), tocopherols (0.002–0.05%), phosphatides (0.1–0.5), serotonin amides (0.03–1%), and chlorogenic acid (0.010%) [5,6,7,19,20]. Coffee oil differentiates from other edible vegetable oils due to the presence of cafestol and kahweol diterpenes, found exclusively in Rubiaceae species with antioxidant, anti-inflammatory, and antitumor properties, consolidating its potential in food, cosmetic, and pharmaceutical applications [6,7,21,22,23]. Serotonin amides are also found in almonds, Brazil nuts, cocoa, hazelnuts, and walnuts. They maintain skin elasticity and exhibit anti-inflammatory, antinociceptive, antioxidant, and neuroprotective activities [24,25]. Chlorogenic acids are recognized for their antioxidant, anti-inflammatory, hepatoprotective, wound-healing, antimutagenic, anticarcinogenic, antiobesity, neuroprotective, and antimicrobial actions [13]. Ester derivatives of linoleic (C18:2; 43–45%), palmitic (C16:0; 32–33%), oleic (C18:1; 9–10%), and stearic (C18:0; 8%) acids reinforce coffee oil’s functionality and biological benefits [6]. Other components are common to other vegetable oils. In addition to these health benefits, coffee lipids influence the sensory attributes of the beverage, such as flavor, body, and creaminess [26,27,28].

In cosmetics, green coffee oil is valued for its antioxidant and moisturizing properties [29], besides its ability to prevent photoaging [30]. Studies demonstrate its effectiveness in protecting against UV-B radiation without cytotoxic effects, making it promising for sunscreen formulations and skin care products [31,32]. Interest in green coffee oil also extends to its industrial potential, especially in the sustainable extraction of its bioactive constituents, which increases its relevance in advanced formulations [33].

Coffee oil can be extracted from the raw bean by pressing, maceration, microwave extraction, ultrasound, accelerated solvent, supercritical fluid, and Soxhlet (Table 1). The industry widely uses the former, and the latter is used in laboratories [34] to correlate oil yield and composition with coffee plant responses to abiotic stress cultivation, climate change, plant–microbiome interactions, plant nutrition, genetic improvement, and plant health [35].

From the data presented in Table 1, it can be observed that the Soxhlet extraction method presents the highest extraction yields (6.4–16.4%). However, its limitation is its long operating time (4–16 h) [48]. Microwave, ultrasonic, accelerated solvent, and supercritical fluid techniques present intermediate values, between 5.9 and 7.6%, 9.1%, 6.3 and 9.8%, and 6.5 and 14.0%, respectively. Meanwhile, extraction by press offers the lowest percentages, ranging from 2.6 to 6.3% (Table 1). Although extraction with supercritical fluid, when the fluid is recycled, is considered a more sustainable alternative, hexane-based methods showed higher extraction percentages (Table 1), demonstrating their efficiency in such conditions.

The greater employability of Soxhlet extraction is due to the higher yield obtained over other techniques and the relatively low cost, being the method recommended by the AOAC (Association of Official Analytical Chemists) since 1965 for the determination of the total lipid content and/or its components from plant or animal matrices [23,37,49,50].

For laboratory-scale work, Soxhlet extraction is the most suitable. This requires a heating mantle for a distillation flask coupled to an extraction chamber (Soxhlet) followed by a condenser [51]. The distillation flask accommodates the extracting solvent (≈300 mL), usually n-hexane, chosen because it is a volatile solvent (boiling point at 68.8 °C), low cost (USD 104.34 L^−1^), and is easy to obtain due to its petrochemical origin [52]; however, it has a toxicity level of 5, is non-renewable and has a high environmental impact [52,53]. This remains heated at boiling temperature for long periods (up to 16 h), then is evaporated and condensed inside the Soxhlet extractor, which contains a cartridge with the crushed sample (≈30 g) and a siphon for removing the extract into the distillation flask, where the solvent is evaporated again to resume the extraction cycle and solvent recovery [39,49,53,54,55,56]. The reflux process in the condenser results in the disposal of 90 L of water for every 1 h of use, that is, 1440 L of water for the 16 h extraction procedure, which over a prolonged time can also lead to the thermal degradation of thermolabile compounds present in the lipid fraction of coffee [26,57]. Therefore, the search for sustainable alternatives is necessary, aiming to reduce energy consumption (3 kWh), solvent amount, and number of samples required [58]. Furthermore, a new method should allow multiple simultaneous extractions, reduce or eliminate the use of water for reflux, and favor automation.

## 2. Results

### 2.1. Lipid Extraction from Green Coffee Beans by Pressing

A sample of raw Arabica coffee beans was pressed using an Expeller screw press, obtaining an oil yield of 3.35% m/m, a value consistent with that found in the literature (Table 1).

### 2.2. Lipid Extraction from Green Coffee Beans by Soxhlet

The official GSLS and AOAC methods are commonly used in lipid extraction from coffee samples, using Soxhlet apparatus for 4 h or 16 h, respectively [39,49]. However, previous studies have shown that these extraction times result in equivalent raw coffee oil yields [26]. For this reason, the 4 h period was adopted to evaluate the lipid content in the sample, with the experiment being conducted in triplicate. Table 2 shows the contents obtained, whose average percentage was 11.56%, a value consistent with literature data, which ranges from 7 to 17% m/m for raw Arabica coffee beans (Table 2) [5,6,19,26].

### 2.3. Lipid Extraction from Green Coffee Beans by Direct Hot Solid–Liquid Extraction (DH-SLE)

#### 2.3.1. Experimental Design

In order to find an alternative to Soxhlet extraction, a new methodology was proposed, for which a smaller quantity of sample and solvent are necessary due to the small quantity of samples available in certain situations of scarce production or rare cultivars and also due to the degree of toxicity of the n-hexane solvent. For these reasons, a DH-SLE was evaluated regarding the recovery of the extracted oil. For this, a 3^3^ full factorial experimental design was used; varying temperature (*T*, °C), time (*t*, min), and the ratio between solvent volume and the mass of ground raw coffee beans (*R*, mL g^−1^) were considered in the design as independent variables, with the response obtained as a function of the oil extraction yield (%) as the dependent variable. In total, 30 experiments were carried out, 27 for the planning and 3 replicates at the central point. The response variables and levels are presented in Table 3, while the experimental matrix containing the results obtained is shown in Table 4.

The experimental data were fitted to the proposed model by analysis of variance (ANOVA) (Table S1). The model was validated by the *F* test (*F_Calculated_* > *F_Tabled_*) obtaining a coefficient of determination of 0.9233 and reproducibility between the central point equal to 2.54% (Table 4: experiments 28–30). The highest yield was identified in experiment 25 (11.64%), which presented the highest values of the variables *T*, *R*, and *t*, and the yield was similar to that found using Soxhlet extraction (Table 2: 11.56%).

The lack of model fit was not significant (*p*-value = 0.6277 > 0.05) (Table S1), indicating that the presence of linear and quadratic terms and the absence of interaction terms enabled model fit. Equation (1) presents the polynomial that describes the experiment, and the coefficients of each term are found in Table S2. Again, the linear factors for *R*, *T*, and *t*, followed by the quadratic factor for *R* were considered significant for describing the experiment, presenting a *p*-value < 0.05.

(1) $$\gamma =10.74+0.75R+0.40T+0.28t-{0.48R}^{2}-{0.12t}^{2}-{0.10T}^{2},$$

Considering Equation (1), it follows that the response surface is concave, due to the quadratic terms of *R*, *t*, and *T* being negative. The only significant quadratic term is *R* (*p*-value ≈ 0.0154 < 0.05), suggesting that small variations in this variable result in substantial changes in the dependent variable. Similarly, given the impact of *R* on linear terms, this stands out as the most significant.

**Figure 1:**
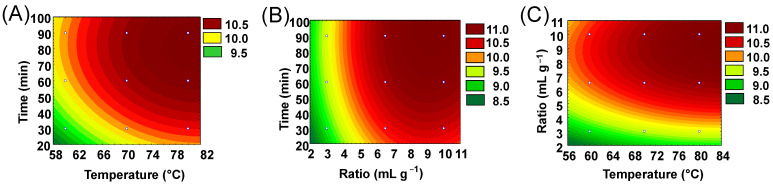
Contour lines illustrate the effect of the variables temperature (*T*), ratio (*R*), and time (*t*) on lipid yield. (**A**) Response surface for time (min) vs. temperature (°C); (**B**) Response surface for time (min) vs. ratio (mL g^−1^); (**C**) Response surface for ratio (mL g^−1^) vs. temperature (°C).

**Figure 2:**
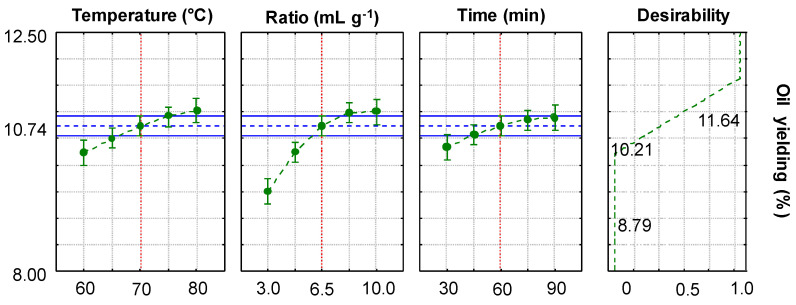
Profile of predicted and desirable values for lipid extraction. The red line represents the mean values used for each variable, the green points correspond to the experimentally obtained data, the dashed blue line represents the extraction for the central points, and the filled blue line covers points around this average extraction. The dashed green curve in the desirability graph shows that higher oil yields are achieved by using the highest values for each parameter.

The model also presented constant variance of the residuals (Figure S1) and adequate adjustment according to the graph of observed versus predicted values (Figure S2), helping to validate the mathematical model that describes the experimental results. The condition with the lowest values of *T*, *R*, and *t* resulted in the lowest extraction yield (Table 4: experiment 1). Thus, according to the experimental model, the increase in all variables positively affects the extraction yield of the procedure. These observations are in agreement with the contour lines (Figure 1) that also indicate the upper axial points (Table 3: level +1) as the best condition for carrying out the extraction: 80 °C, 10 mL g^−1^, and 90 min. The best extraction condition is also obtained by the desirability function, indicated by the curves of the graphs in Figure 2 and the value of 0.93637 for the function, corresponding to 11.64% of the extraction yield.

**Figure 3:**
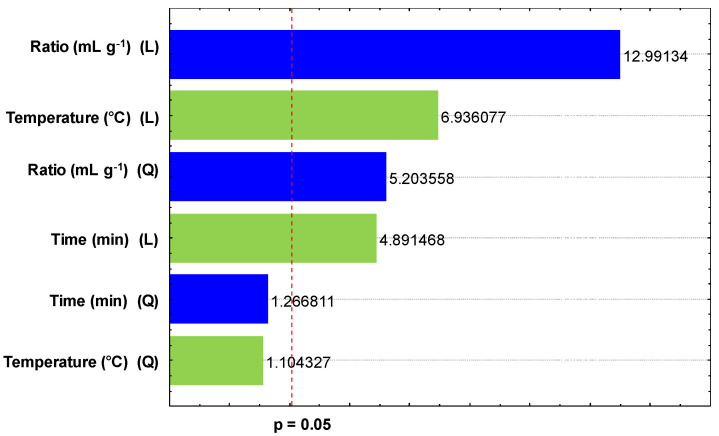
Pareto chart for the estimated effects on DH-SLE of lipids.

The Pareto chart (Figure 3) shows the linear terms (L) of the *R* and *T* as the most important for extraction, followed by the quadratic term (Q) of the *R* and the linear term of *t*. The Pareto chart shows agreement with the *F*-test (Table S2), with the results of the predicted and desirable values for extraction (Figure 2), and with the response surfaces presented in Figure 1.

Regarding the variables evaluated, it can be stated that higher *T* values result in a positive effect on lipid extraction by reducing the viscosity of the solvent, facilitating its permeation into the bean interstices, thus increasing the solubility of lipids in the solvent and, therefore, mass transfer. Temperatures higher than 80 °C were not considered in the experiment due to the thermolability of the diterpenes cafestol and kahweol, coffee lipid components [20,26].

A greater quantity of solvent concerning the coffee mass (*R* = 10) also provided a greater extraction yield. Lower *R* values tend to saturate the solvent, once it exceeds its solvation capacity, which was observed through the lowest ratios tested (Table 4, Figure 1 and Figure 2). Based on these results, it was decided to use a more diluted extractive medium, with *R* = 10 under heating at 80 °C for the DH-SLE of coffee lipids.

The level curves demonstrate an optimal region for the maximum points of each variable (Figure 1), just as the graphs of the desirability function show a certain plateau for the variables (Figure 2). This condition demonstrates that the increase in variables outside the studied ranges may not be significant in terms of the extraction yield. For the reasons stated above, it was not desirable to increase *T* or *R*; however, an investigation into the time variable is appropriate.

#### 2.3.2. Optimization of DH-SLE and Validation Against Soxhlet Extraction

A new experiment was performed to verify whether the 90-min extraction was exhaustive by comparing it to longer extraction times.. Thus, a univariate analysis was performed by extending the extraction time from 90 min to 120 and 150 min. Table 5 presents these results, with equivalent extraction yields observed as a function of the average values and the respective coefficients of variation.

This equivalence in the results is confirmed by the analysis of variance (Table S3), whose expected *F*-value (2.42) was greater than the *F_calculated_* (0.5497), indicating no significant difference between the data, ensuring the use of 90 min for lipid extraction. Times of 30 and 60 min were previously evaluated (Table 4: Experiments 26 and 27) and showed lower yields, i.e., 10.64 and 11.15%, as already discussed in the previous section.

Finally, an extraction time of 90 min and a temperature of 80 °C with ratio of 10 mL g^−1^ was chosen as the optimized method for the lipid yield determination of raw Arabica coffee bean samples.

When comparing the average lipid extraction percentages obtained by the Soxhlet method (Table 2: 11.56%) with the optimal DH-SLE conditions (Table 5: 11.59%), a notable equivalence is observed, with a difference of only 0.03%. This highlights the effectiveness of the DH-SLE approach over the conventional Soxhlet method. To verify the difference between the methods, an ANOVA was performed between the data (Table S4), and the results (*p*-value = 0.8588) suggest that the extraction percentages are statistically equal. Additionally, the *F*-calculated value (0.0360) is smaller than the *F*-critical value (7.7086), reinforcing the conclusion that there are no significant differences between the methods. Thus, the optimized DH-SLE is considered an equivalent alternative to Soxhlet extraction.

#### 2.3.3. Fatty Acid Composition

To compare the fatty acid composition of the oils extracted by pressing, Soxhlet and DH-SLE were subjected to transesterification and analyzed by gas chromatography–mass spectrometry (GC-MS). The analysis of free fatty acids by GC-MS resulted in broad peaks, hence the low peak capacity and multiple coelutions [59]. To solve this problem, the transesterification reaction was carried out to convert free fatty acids and all their ester derivatives (mono, di, and triacylglycerols and diterpene esters of cafestol and kahweol, among others) into their respective alcohols and fatty acid methyl esters (FAMEs) [59,60,61]. The reaction performs this conversion through excess methanol in the presence of a catalyst, without altering the fatty acid composition [59,60,61]. In this way, FAMEs are determined with symmetrical and fully captured peaks, which elute at a lower temperature than their source molecules, resulting in chromatograms with greater peak capacity without thermally stressing the capillary column [59]. The reaction occurred with 100% conversion, with the FAME profile and its respective levels being sufficient for comparison purposes [59]. Figure S3 shows the three GC-MS chromatograms, and Table 6 shows the percentage (% m/m) of the chemical composition obtained through area normalization. From these results, we can observe observe chromatograms with notable similarities in composition, suggesting that the extraction techniques employed resulted in equivalent profiles, formed mainly by linoleic (36–37%), palmitic (32–34%), oleic (10–11%), and stearic (10–11%) acids (Table 6). These results are in agreement with those found in the literature, which presents similar values for these fatty acids: linoleic (40–54%), palmitic (25–35%), oleic (6–14%), and stearic (5–7%) [6,62]. Finally, it can be concluded that DH-SLE proved to be promising due to a composition of FAMEs similar to the other techniques evaluated, in addition to achieving an extraction percentage equivalent to the conventional Soxhlet technique, widely used in plant oil extraction.

#### 2.3.4. Compliance with the United Nations Sustainable Development Goals

In addition to the equivalence in oil extraction efficiency and its fatty acid composition, DH-SLE is a safer and more sustainable procedure, as it uses only 2 mL of solvent compared to the 300 mL required for Soxhlet extraction—due to the physical structure of the glassware that requires a larger number of samples. Another advantage is that the method requires less extraction time (1.5 h), while Soxhlet requires 4 to 16 h, requiring greater energy expenditure (3.0 kWh, compared to 1.5 kWh for DH-SLE). In addition to these issues, another point to be raised is the high level of water consumption required to cool the condenser used in Soxhlet extraction. It has been estimated that the use and disposal of 90 L of water for every 1 h of condenser use and the proposed method does not use water for cooling. The amount of sample is another important factor, since there may be little availability, reducing costs and time for grinding the material (0.2 g of sample versus 30 g for Soxhlet). Furthermore, the procedure developed allows 24 simultaneous extractions to be carried out on a heating plate, compared to only 1 extraction per Soxhlet, although there are systems that allow 6 or more Soxhlet extractors to be used simultaneously. It is important to highlight that DH-SLE can be easily applied on a large scale and is easier to automate. These advantages are summarized in Table 7.

These gains are in line with the United Nations Sustainable Development Goals (SDGs): (Goal 3.9) reducing exposure to chemicals and their impacts on human health; (Goal 6.3) eliminate wastewater discharge and (Goal 6.4) increase water use efficiency; (Goal 9.1) reliable, sustainable, and (Goal 9.2–3) inclusive development with (Goal 9.4) environmentally sound process; (Goal 11.6) reduction in negative environmental impact, (Goal 12) ensuring consumption patterns and sustainable management of chemical products and their waste, reducing their generation and release into the environment and their impacts on human health through prevention, reduction, and possible recycling and reuse of the solvent; and (Goal 14) conservation and sustainable use of water [63].

#### 2.3.5. Return on Investment (ROI) Analysis

The costs associated with extractions performed by DH-SLE and Soxhlet are detailed in Table 8. DH-SLE showed significant reductions in all items evaluated, demonstrating its economic efficiency concerning Soxhlet extraction.

ROI analysis indicated a 98.79% savings in total cost per extraction when replacing Soxhlet with DH-SLE. This saving is attributed in particular to the reduction in solvent, energy, and water consumption, in addition to the smaller amount of sample required for the DH-SLE method.

The results reinforce the viability of the DH-SLE method as a more sustainable and economical alternative to Soxhlet extraction, with the potential for large-scale applications. In addition to its resource efficiency, the method offers additional advantages, such as reduced extraction time and the possibility of automation.

## 3. Materials and Methods

### 3.1. Chemicals and Materials

Raw Arabica coffee beans were provided by the Department of Phytopathology of the Federal University of Viçosa (UFV), MG, Brazil (Coffee Nursery, 20°44′53″ S, 42°15′106″ W, 667 m altitude; harvested in June 2023). These were dried on a suspended patio until they reached a humidity level of 11%. Some of the dried beans were pressed in an infinite screw expeller press by the company COOXUPÉ (Regional Coffee Growers’ Cooperative, Guaxupé, MG, Brazil). A second fraction of the same beans was ground (SL 38 mill, SOLAB, Piracicaba, São Paulo, SP, Brazil) and sieved through a sieve (Model 15P4040.08.C, Bronzinox, São Paulo, SP, Brazil) to obtain particles measuring 0.686 to 1.651 mm. These ground beans were used to evaluate the solid–liquid extraction by Soxhlet (item 2.2) and direct hot extraction (item 2.3).

Methanol and n-hexane (HPLC grade) were purchased from Sigma-Aldrich (HPLC grade, São Paulo, Brazil) and potassium hydroxide (KOH) from Vetec (Duque de Caxias, RJ, Brazil). A mixture of 37 fatty acid methyl esters (FAME) was purchased from Supelco (cod. number 47885-U, Sigma-Aldrich, St. Louis, MO, USA).

### 3.2. Solid–Liquid Extraction by Soxhlet

Thirty grams of sample were used for Soxhlet extraction with 300 mL of *n*-hexane for 4 h (4 cycles/h) [49]. After the extraction and cooling of the system, the extracts were concentrated in a rotary evaporator (RV 3, IKA, Campinas, SP, Brazil) under reduced pressure until constant mass, which was used to determine the lipid content (Equation (2)).

(2) $$Extraction\;yield\;\left(\%\right)=\frac{Mass\;of\;extracted\;oil}{Mass\;of\;ground\;coffee\;beans}\times 100\%,$$

### 3.3. Direct Hot Solid–Liquid Extraction (DH-SLE)

**Figure 4:**
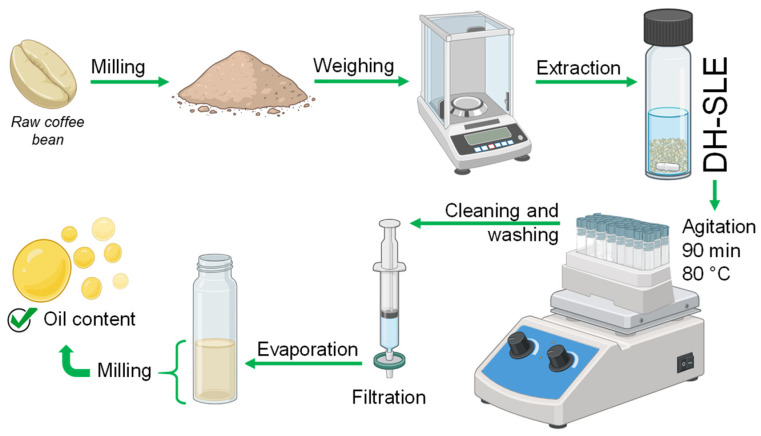
Experimental scheme of the DH-SLE procedure.

Two hundred milligrams of crushed sample were weighed into a screw-top vial (4 mL), subsequently added a magnetic bar (3 mm × 10 mm) and 2 mL of n-hexane. The vial was hermetically closed with a screw cap containing an internal PTFE seal and subjected to direct extraction at 80 °C for 90 min under vigorous stirring. The resulting suspension was collected and filtered to remove the suspended solids using a syringe filter (Teflon membrane, 13 mm × 0.45 μm, Labquip Technologies, Melbourne, Australia). The solid residue was washed twice with 2 mL of hexane and the extract was also filtered and added to the final extract, which was evaporated for gravimetric analysis of lipid content (Equation (2)). A schematic of the procedure is shown in Figure 4.

### 3.4. Transesterification of Oils

Fatty acids were converted into FAMEs in accordance with the International Olive Council guidelines [64]. Briefly, 100 mg of the oil sample was transferred to a 5 mL vial, to which 2 mL of hexane and 0.5 mL of 2 mol L^−1^ KOH prepared in methanol were added. The vial was hermetically sealed and shaken vigorously for 30 s. After resting, two clear phases were formed, with the upper phase consisting of FAMEs, which was filtered through a syringe filter (Teflon membrane, 13 mm × 0.45 μm, Labquip Technologies, Melbourne, Australia) and transferred to a 2 mL vial for immediate analysis by gas chromatography.

### 3.5. FAME Analysis

An Agilent Technologies, Inc. (Palo Alto, CA, USA) model 7820 gas chromatograph (GC) equipped with a G4513A auto-sampler and coupled to an Agilent quadrupole mass spectrometer (MSD 5973 Network) was used. Helium carrier gas (99.9992% purity) was implemented at 1.5 mL min^−1^ in the constant flow mode. The DB-Wax capillary column (polyethylene glycol (PEG) stationary phase consisted of 30 m length, 0.25 mm i.d., and a film thickness of 0.25 μm, J&W Scientific, Agilent). An injector with a flow split (1:20) at 220 °C and an injection volume of 1.0 µL. The GC oven temperature program was as follows: 50 °C (hold 1 min) to 150 °C at 10 °C min^−1^, then 150 to 240 °C (at 3 °C min^−1^), and hold at 240 °C for 5 min.

Mass spectrometer (MS) operating conditions were as follows: ion source temperature, 230 °C; interface temperature, 250 °C; quadrupole temperature, 200 °C; and ionization voltage, 70 eV. Mass spectra obtained in scanning mode (50–500 Da). Data acquisition and processing were performed using MassHunter GC/MS Acquisition B.07.04.2260 software (Agilent Technologies, Inc.). FAMEs were identified through a comparison of their retention time with authentic standards and also through comparison with NIST/EPA/NIH Mass Spectral Library databases (2.2 version, 2014, Standard Reference Data Program of the NIST, USA). All runs were performed in triplicate. 

### 3.6. Statistical Analysis

Experimental planning and statistical analysis were performed using Statistic software version 7.0 (Statsoft, Inc., Tulsa, OK, USA), taking 5% as the significance level. A quadratic model under a 3^3^ full factorial experimental design was used to describe the lipid content as a function of the three factors studied: the ratio between solvent volume and mass of ground green coffee beans (*R*, mL g^−1^), the temperature (*T*, °C) and the time (*t*, min) of extraction (Section 2.3).

### 3.7. Return on Investment Analysis

Return on investment (ROI) analysis was determined to quantify the savings provided by replacing Soxhlet extraction with DH-SLE (Equation (3)) [65,66]. The analysis considered (i) the consumption of the solvent used (hexane: 104.34 USD/L), (ii) the consumption of electrical energy (0.12 USD/kWh), and (iii) the consumption of water.

(3) $$ROI(\%)=\left(\frac{\sum Soxhlet\;cost-\sum DH-SLE\;cost}{\sum Soxhlet\;cost}\right)\times 100$$

## 4. Conclusions

The proposed DH-SLE methodology demonstrated an extraction yield equivalent to Soxhlet extraction after implementing a 3^3^ full factorial experimental design, optimizing the parameters of time, temperature, and the ratio between raw coffee bean’s solvent volume and mass. The best performance was achieved with 90 min, at 80 °C, and a ratio of 10 mL g^−1^, resulting in an extraction of 11.6% m/m of Arabica coffee oil. In addition to the equivalence in extraction efficiency and fatty acid composition, DH-SLE is a sustainable approach, substantially reducing the sampling scale and the volume of solvent required, using only 2 mL of solvent, compared to the 300 mL used in Soxhlet extraction. In addition, the method reduces the extraction time to only 1.5 h, while Soxhlet requires 4 to 16 h, resulting in an energy consumption of 1.5 kWh, compared to 3.0 kWh for Soxhlet. Another relevant aspect is the elimination of water consumption for cooling, estimated at 90 L/h in Soxhlet. The amount of sample required is also considerably smaller (0.2 g for DH-SLE versus 30 g for Soxhlet), reducing costs and preparation time. Return on investment analysis indicated 99% savings in total cost per extraction when replacing Soxhlet with DH-SLE. The system developed for DH-SLE allows 24 simultaneous extractions, in contrast to Soxhlet, which generally performs one extraction at a time, although there are systems for multiple Soxhlets. This method also has additional advantages, such as the provision of automation and scalability for industrial applications. Therefore, DH-SLE represents a significant advance in the extraction of lipids for analytical purposes, promoting a more efficient, sustainable, and economical approach, with potential for application in other plant matrices. In addition, the method is aligned with the United Nations Sustainable Development Goals (SDGs 3, 6, 9, 11 and 12), reinforcing its positive impact on sustainable development. 

## Figures and Tables

**Table 1:** Lipid extraction methods in green coffee beans (GCBs).

Extraction Technique	Procedure Details	Yield (%)	Reference
Soxhlet	20 g of GCB 200 mL hexane 6 h of extraction under reflux	11.4	[36]
	2–5 g of GCB 250–300 mL petroleum ether 8–16 h of extraction under reflux	Not informed	[37]
	7.5 g of GCB 30 mL petroleum ether 4 h of extraction under reflux	7.5–9.5	[38]
	30 g of GCB 300 mL hexane 4–16 h of extraction under reflux	10.4–10.5	[26]
	7.5 g of GCB 30 mL petroleum ether 4 h of extraction under reflux	Not informed	[39]
	50 g of GCB 250 mL hexane 8 h of extraction under reflux	15	[40]
	50 g of GCB 100 mL hexane 7 h of extraction under reflux	8.5	[41]
	2 g of GCB 10 mL solvent 3 h of extraction under reflux	8.3 (acetone) 11.7 (ethanol) 6.4 (ethyl acetate) 8.8 (hexane) 10.2 (isopropanol) 7.7 (petroleum ether)	[42]
	25 g of GCB 400 mL of petroleum ether 4 h of extraction under reflux	9.5	[23]
	2 g of GCB petroleum ether 6 h of extraction under reflux	14.0	[43]
	Industrial Soxhlet 25 kg GCB Hexane 16 h of extraction under reflux	10–12	[44]
	20 g GCB 150 mL hexane 4 h of extraction under reflux	9.1–16.4	[7]
Microwave assisted extraction	2 g of GCB 8 mL petroleum ether 45 °C; 10 min; 600 rpm	5.9–7.6	[38]
	10 g of GCB 100 mL ethanol; 60 °C; 30 min; 200 W	9.3	[45]
Ultrasonic extraction	10 g of GCB 300 mL ethanol 35 °C; 40 kHz; 50 min; 50 W	9.1	[45]
Microwave assisted ultrasonic extraction	3.6 g of GCB 100 mL ethanol 60 °C; 350 W; 10 min	10.6	[45]
Accelerated solvent extraction	22 g of GCB 30 mL ethanol 3 cycles; 10.3 Mpa	6.6–9.8	[46]
	20 g of GCB 40 mL ethanol 100 °C; 100 bar; 30 min	6.3	[45]
CO_2_ Supercritical Fluid Extraction	200 mg of GCB or RCB 60–90 °C; 235–380 bar; 25 min; 1.5 mL min^−1^ of CO_2_	10.3–14.0	[36]
	90 g of GCB; 90 °C; 300 bar; 695.42 kg/m^3^; 6 h; CO_2_ at 5 g/min	6.5	[47]
	90 g of GCB 70 °C; 300 bar 6 h; CO_2_ at 5 g/min	8.8	[46]
Expeller screw press	350 g of GCB Continuous screw press 5 mm outlet, 18 rpm	5.3–7.0	[7]
	1 kg of GCB 0.67 to 1.2 kg/h 18 rpm	2.6–6.3	[23]

**Table 2:** Lipid yield (%) of Soxhlet extraction from raw coffee beans.

Experiment	Yield (%)
Replicate 1	11.54
Replicate 2	11.50
Replicate 3	11.65
Mean	11.56
Standard deviation	0.08
Coefficient of variation (%)	0.67

**Table 3:** Variable levels in the full factorial experimental design.

Factor	Levels		
	Low (−1)	Center (0)	High (+1)
*T*, temperature (°C)	60	70	80
*R*, the ratio between solvent volume and the mass of ground raw coffee beans	3:1 (2 mL:0.667 g^−1^)	6.5:1 (2 mL:0.308 g^−1^)	10:1 (2 mL:0.200 g^−1^)
*t*, time (min)	30	60	90

**Table 4:** Experimental design and response values.

Experiment	*T* (°C)	*R* (mL g^−1^)	*t* (min)	Coffee Oil Yield (%)
1	60	3.0	30	8.79
2	60	3.0	90	9.21
3	60	3.0	60	8.97
4	60	6.5	90	10.27
5	60	6.5	60	10.41
6	60	6.5	30	10.00
7	60	10.0	60	10.24
8	60	10.0	30	9.76
9	60	10.0	90	10.81
10	70	3.0	90	9.64
11	70	3.0	60	9.80
12	70	3.0	30	8.82
13	70	6.5	60	10.79
14	70	6.5	30	10.41
15	70	6.5	90	11.09
16	70	10.0	30	10.93
17	70	10.0	90	11.18
18	70	10.0	60	10.99
19	80	3.0	60	10.19
20	80	3.0	30	9.29
21	80	3.0	90	9.53
22	80	6.5	30	10.58
23	80	6.5	90	11.19
24	80	6.5	60	11.17
25	80	10.0	90	11.64
26	80	10.0	60	11.15
27	80	10.0	30	10.64
28	70	6.5	60	10.73
29	70	6.5	60	10.64
30	70	6.5	60	10.23
Average value (Exp. 28–30)				10.53
Standard deviation (Exp. 28–30)				0.27
Coefficient of variation (%; Exp. 28–30)				2.54

*T*: temperature; *t*: time; *R*: the ratio between solvent volume and the mass of ground coffee beans.

**Table 5:** Coffee oil yield (%) vs. extraction time when *R* = 10 mL g^−1^ and *T* = 80 °C.

Coffee Oil Yield (%)	Extraction Time (min)		
	90	120	150
Replicate 1	11.75	11.73	11.38
Replicate 2	11.74	77.83	11.49
Replicate 3	11.29	11.34	11.50
Average value	11.59	11.63	11.46
Standard deviation	0.26	0.26	0.07
Coefficient of variation (%)	2.27	2.23	0.58

**Table 6:** Percentage composition of coffee oils fatty acids extracted by pressing, Soxhlet (4 h), and DH-SLE (90 min).

Time (min)	Compound Name	Pressing (%)	Soxhlet (%)	DH-SLE (%)
16.208	Myristic acid (C14:0)	0.10 ± 0.04	0.12 ± 0.06	0.07 ± 0.03
20.797	Palmitic acid (C16:0)	32.78 ± 2.14	32.93 ± 2.23	34.14 ± 3.54
25.637	Stearic acid (C18:0)	10.30 ± 0.72	9.95 ± 0.97	9.83 ± 1.26
26.101	Oleic acid (C18:1)	11.90 ± 0.45	10.92 ± 1.13	11.57 ± 0.94
27.383	Linoleic acid (C18:2)	36.97 ± 2.89	37.41 ± 2.73	37.07 ± 1.93
28.790	Linolenic acid (C18:3)	1.85 ± 0.20	1.35 ± 0.13	1.50 ± 0.26
30.593	Arachidic acid (C20:0)	3.97 ± 0.97	4.43 ± 0.27	3.70 ± 0.77
31.022	Gadoleic acid (C20:1)	0.46 ± 0.15	0.45 ± 0.18	0.40 ± 0.13
35.439	Behenic acid (C22:0)	0.92 ± 0.28	1.32 ± 0.42	0.86 ± 0.40
36.452	Erucic acid C22:1)	0.36 ± 0.10	0.67 ± 0.25	0.54 ± 0.13
40.097	Lignoceric acid (C24:0)	0.39 ± 0.13	0.46 ± 0.09	0.33 ± 0.11

**Table 7:** Comparison of the feasibility of lipid extractions in raw coffee via Soxhlet and DH-SLE.

Sample Estimation	Soxhlet	DH-SLE
Sample mass (g)	30	0.2
Volume of *n*-Hexane (mL)	300	2
Extraction time (h)	4–16	1.5
Water consumption per hour (L)	90	0
Energy consumption (kWh)	3.0	1.5
Coffee oil yield (% *w*/*w*)	11.5	11.6

**Table 8:** Comparison of operational costs of lipid extractions in raw coffee via Soxhlet and DH-SLE.

Item	DH-SLE (USD)	Soxhlet (USD)	Economy (USD)
Hexane cost	0.21	31.30	31.09
Energy cost	0.18	0.36	0.18
Water cost	0.00	0.54	0.54
Total by extraction	0.39	32.20	31.81

## Data Availability

Data are contained within the article or Supplementary Materials.

## Supplementary Materials

The following supporting information can be downloaded at: https://www.mdpi.com/article/10.3390/plants14020185/s1. Table S1. Analysis of variance (ANOVA) to validate the mathematical model; Table S2. Estimated effect parameters for DH-SLE; Figure S1. Plot of observed values vs. residuals; Figure S2. Graph of observed values vs. predicted values; Table S3. Analysis of variance for the oil extraction values in Table 5; Table S4. Analysis of variance for the validation values of DH-SLE compared to Soxhlet.
