# Supplementary material for: Direct Hot Solid–Liquid Extraction (DH-SLE): A High-Yield Greener Technique for Lipid Recovery from Coffee Beans
Source: Plants (Basel). 2025 Jan 11;14(2):185. doi: 10.3390/plants14020185 (PMC11768105; doi:10.3390/plants14020185)
Supplement: Supplementary file 1 [file plants-14-00185-s001.zip › plants-3347689-supplementary.pdf]

Supplementary Materials – Plants

Special Issue " *Valuable Phytochemicals: Extraction Technologies, Analyte Isolation, Bioactive and Nutritional Properties*"

Direct hot solid-liquid extraction (DH-SLE): A high-yield greener technique for lipid recovery from coffee beans

Daliane Cláudia de Faria, Maria Eliana Lopes Ribeiro de Queiroz, Fábio Junior Moreira Novaes

Universidade Federal de Viçosa, Departamento de Química, Avenida Peter Henry Rolfs, s/n, Campus Universitário, Viçosa, MG 36570-900, Brazil; daliane.faria@ufv.br (D.C.d.F.); meliana@ufv.br (M.E.L.R.Q.)

*Correspondence: fabio.novaes@ufv.br (Fábio Junior M. Novaes); Tel.: +55 31 3612-6635

CONTENTS:

Table S1. Analysis of variance (ANOVA) to validate the mathematical model. 2

Table S2. Estimated effect parameters for DH-SLE. 2

Figure S1. Plot of observed values *vs* residuals. 3

Figure S2. Graph of observed values *vs* predicted values. 3

Table S3. Analysis of variance for the oil extraction values in Table 5. 4

Table S4. Analysis of variance for the validation values of DH-SLE compared to Soxhlet. 4

Table S1. Analysis of variance (ANOVA) to validate the mathematical model.

Factor	Quadratic sum	Degree of Freedom	Quadratic Mean	$F_{Calculated}$	$F_{Tabulated}$	*p*- Value
Temperature (*T*, L)	2.8575	1	2.8575	44.3063	2.42	0.0069
Temperature (*T*, Q)	0.0724	1	0.0724	1.2131	2.42	0.3670
Ratio (*R*, L)	10.0245	1	10.0245	155.4339	2.42	0.0011
Ratio (*R*, Q)	1.6083	1	1.6082	24.9367	2.42	0.0155
Time (*t*, L)	1.4211	1	1.4211	22.0352	2.42	0.0183
Time (*t*, Q)	0.0953	1	0.0953	1.4780	2.42	0.3110
Lack of Adjustment	1.1726	20	0.0586	0.9091	2.42	0.6277
Pure Error	0.1935	3	0.0594			
Total	17.8195	29				

Table S2. Estimated effect parameters for DH-SLE.

Factor	Effect	Coefficient	Standard error of the coefficient	*p*-value
Mean/Interc.	10.7361	10.7362	0.0970	<0.0000
Ratio (R, L)	1.4925	0.7463	0.0599	0.0011
Temperature (T, L)	0.7969	0.3984	0.0599	0.0069
Time (t, L)	0.5620	0.2810	0.0599	0.0183
Ratio (R, Q)	-0.9686	-0.4843	0.0970	0.0154
Time (t, Q)	-0.2358	-0.1179	0.0970	0.3110
Temperature (T, Q)	-0.2056	-0.1028	0.0970	0.3670

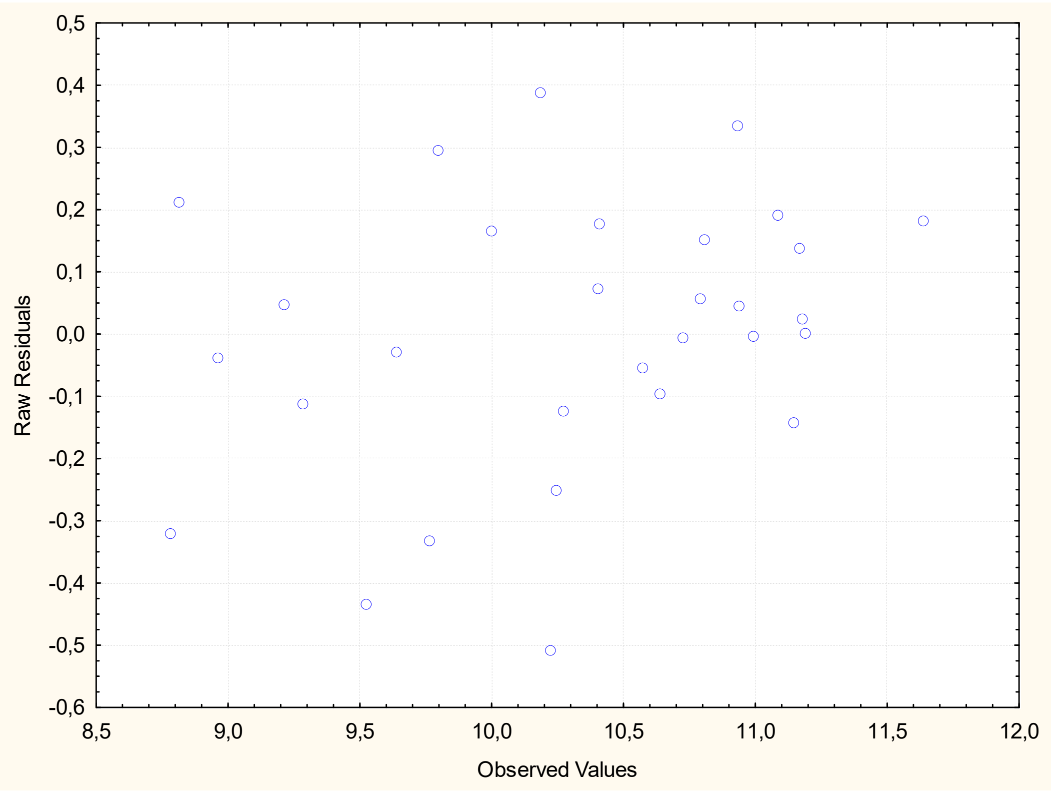

Figure S1. Plot of observed values *vs* residuals.

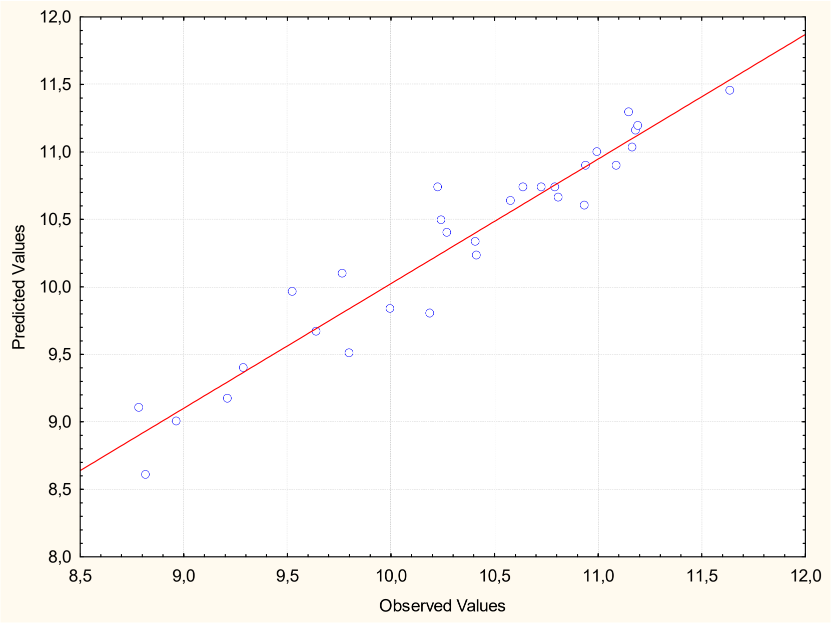

Figure S2. Graph of observed values *vs* predicted values.

Table S3. Analysis of variance for the oil extraction values in Table 5.

Factor	Quadratic Sum	Degree of Freedom	Quadratic Mean	$F_{Calculated}$	$F_{Tabulated}$	*p*-value
Between groups	0.0515	2	0.0257	0.5497	2.42	0.6037
Within groups	0.281	6	0.0468			
Total	0.3325	8				

Table S4. Analysis of variance for the validation values of DH-SLE compared to Soxhlet.

Factor	Quadratic Sum	Degree of Freedom	Quadratic Mean	$F_{Calculated}$	$F_{Tabulated}$	*p*-value
Between groups	0.0014	1	0.0014	0.0360	7.7086	0.8588
Within groups	0.1501	4	0.0375			
Total	0.1515	5				
